# Four methylation‐driven genes may be prognostic biomarkers in clear cell renal carcinoma

**DOI:** 10.1002/ctm2.45

**Published:** 2020-06-04

**Authors:** Hang Yin, Haiyang Zhang, Xiaoyuan Wang, Qingyong Xu

**Affiliations:** ^1^ Department of Radiation Oncology Harbin Medical University Cancer Hospital Harbin China; ^2^ Key Laboratory of Hepatosplenic Surgery Ministry of Education The First Affiliated Hospital of Harbin Medical University Harbin China; ^3^ Department of Oncology Harbin Medical University Cancer Hospital Harbin China

Dear Editor,

Clear cell renal carcinoma (ccRCC) is one of the most common urinary system carcinomas with a 5‐year survival about 44–69%.^1^ There is an urgent need to explore effective biomarkers to predict the survival of patients with ccRCC. Epigenetic mechanisms as an important part in the development of tumorigenesis mainly include heritable and reversible DNA methylation. DNA methylation could negatively regulate gene expression in the development of tumors, and be also used as biomarkers for prognostic prediction. It is important and urgent to explore the abnormity of DNA methylation‐associated genes. Recently, there are more and more studies based on the Cancer Genome Atlas (TCGA) database with large amounts of genetic and clinical profiles.^2^ In addition, MethylMix is used to identify methylation status according to the β model.^3^

The present study aims at understanding gene expression and methylation as well as the association with clinical information in patients with ccRCC and screening the methylation gene‐based biomarkers for prognostic prediction. We obtained 524 complete clinical data and mRNA data of patients with ccRCC (539 cancer and 72 para‐carcinoma tissues). Note that 2332 differential expression genes were considered as discriminatively expressed (the absolute value of fold changes is > 2, and the value of false discovery rate is < 0.01). The methylated data were obtained from TCGA and Human Methylation 450k Illumina Infinium methylation arrays. The methylated data included 160 normal samples and 325 tumor samples. The aberrant methylation and expression of genes in ccRCC were explored by LIMMA package. Then the aberrant genes were further explored by the MethylMix package (|logFC| > 0, *P* < .05, |Cor| > 0.3). We also explored 168 methylation‐driven genes (Figure 1A,B), of which 75 genes (44.6%) were hypermethylated and the other genes were hypomethylated. The expression of genes with significant difference in tumor samples as compared with the normal were investigated, of which 51 abnormally methylated and differentially expressed genes in ccRCC (Figure 1C).

**FIGURE 1:**
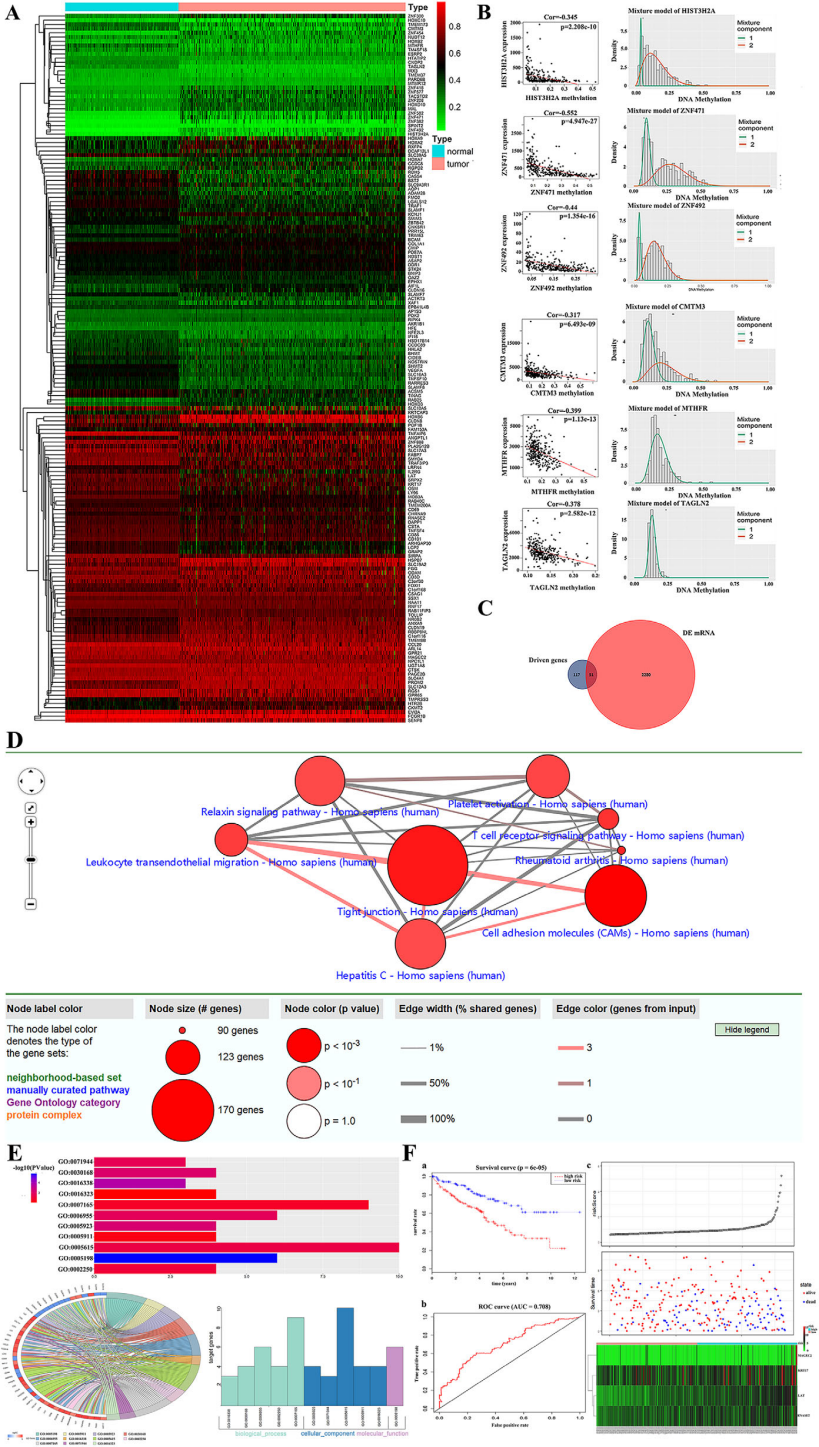
A, Heat maps of ccRCC‐related aberrant methylated genes. The color from green to red shows a trend from low expression to high expression. ccRCC: clear cell renal carcinoma. B, The top hypermethylated and top hypomethylated genes of tumor samples compared with normal samples. The *x*‐axis is the methylation degree, the *y*‐axis is the number of methylation samples, the histogram represents the methylation distribution of cancer group, and the curve is the simulated trend curve of the methylation distribution of cancer group. The black horizontal line above the figure is the methylation level distribution of the normal sample. C, Fifty‐one abnormally methylated and differentially expressed genes in ccRCC. D, The KEGG pathways of these genes were explored by ConsensusPathDB. The enriched pathways were cell adhesion molecules, Hepatitis C, leukocyte transendothelial migration, platelet activation, relaxin signaling, rheumatoid arthritis, T‐cell receptor signaling, and tight junction. E, The function of these genes was explored by DAVID. GO:0071944‐cell periphery, GO:0030168‐platelet activation, GO:0016338‐calcium independent cell‐cell adhesion, GO:0016323‐basolateral plasma membrane, GO:0007165‐signal transduction, GO:0006955‐immune response, GO:0005923‐bicellular tight junction, GO:0005911‐cell‐cell junction, GO:0005615‐extracellular space, GO:0005198‐structural molecule activity, and GO:0002250‐adaptive immune response. F, Identification and performance evaluation of the four‐gene signature in the training dataset. a, Kaplan‐Meier survival curve analysis for overall survival of clear cell renal cell carcinoma patients using the 4‐gene signature in the training dataset; b, ROC curve analysis of the 4‐gene signature in the training dataset; c, the distributions of the survival status and expression profiles of the four genes of patients in the training dataset

We explored the function of these genes by the Discovery 6.8 (DAVID) (https://david.ncifcrf.gov/) and constructed KEGG pathways using ConsensusPathDB. The results showed the function of these genes were cell periphery, platelet activation, immune response, cell‐cell junction, and others. The KEGG pathways of these genes were rheumatoid arthritis, platelet activation, hepatitis C, and so on (Figure 1D,E). To analyze the function of abnormally methylated and differentially expressed genes, we calculated the relationship between the 51 genes and overall survival in the training set. The results of univariate cox analysis showed that eight genes were correlated with overall survival (*P* < .05). Of eight genes in multivariate Cox regression, four genes including Linker for Activation of T Cells (LAT), Keratin 17 (KRT17), MAGE Family Member C2 (MAGEC2), and Ribonuclease A Family Member 2 (RNASE2) were independent risk factors effecting existence (*P* < .001; Table S1). The 4‐gene expression‐based survival risk score was calculated as 0.991. We also compared the survival rate between patients with low and high risks according to the risk score and the Kaplan‐Meier curves. The patients with high risk had shorter survival time than those with low risk (*P* < .001). The 5‐year survival times of patients with low and high risks were 75.6 (95% CI = 0.67‐‐0.825) and 50.7 (95% CI = 0.4115‐‐0.625), respectively. The area under ROC curve (AUC) was 0.708 (Figure 1F).

We further evaluated the value of the four target genes in the prognostic significance. The 5‐year survival time of patients with high risk (*n* = 168; 52.7 (95% CI = 0.446‐‐0.624)) was shorter than those with low risk (*n* = 97; 86.2 (95% CI = 0.789‐‐0.942), *P* < .001), respectively. The AUC was 0.732 (Figure 2A). Figure 2B demonstrated the result of the four genes in the entire group. The correlation among gene methylation and expression with patient survival using the survival package of R. KRT17, LAT, MAGEC2, and RNASE2 were significantly correlated with overall survival (*P* < .05, Figure 2C). The correlations between methylation sites and gene expressions were also analyzed. The absolute value of Cor > 0.2 was the cut off value. The expression of LAT, MAGEC2, and RNASE2 were associated with the methylation level of multiple sites (Figure S10). To explore the correlations between methylation and expression, OSRC2 and 786O cells of ccRCC were treated by 5‐Aza (8 µM) to explore the relationship between the blocking of promoter in methylation and expression. The expression of LAT, KRT17, MAGEC2, and RNASE2 genes increased significantly in both cells after the treatment, as compared with P16 and CST6 as controls (Figure 2D).

**FIGURE 2:**
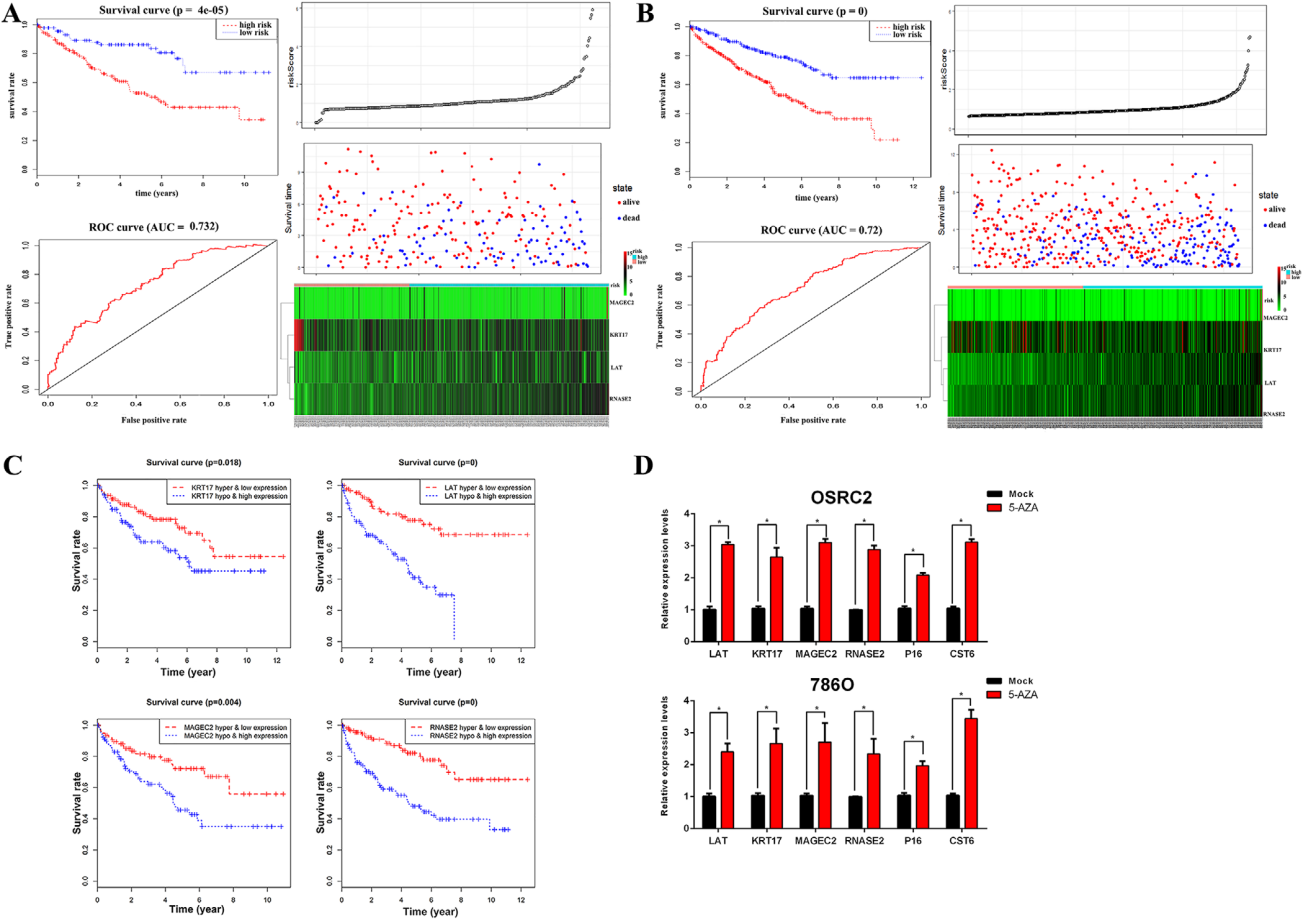
A, Identification and performance evaluation of the 4‐gene signature in the testing dataset. a, Kaplan‐Meier survival curve analysis for overall survival of clear cell renal cell carcinoma patients using the 4‐gene signature in the testing dataset; b, ROC curve analysis of the 4‐gene signature in the testing dataset; c, the distributions of the survival status and expression profiles of the four genes of patients in the testing dataset. B, Identification and performance evaluation of the 4‐gene signature in the entire dataset. a, Kaplan‐Meier survival curve analysis for overall survival of clear cell renal cell carcinoma patients using the 4‐gene signature in the entire dataset; b, ROC curve analysis of the 4‐gene signature in the entire dataset; c, the distributions of the survival status and expression profiles of the four genes of patients in the entire dataset. C, Kaplan–Meier survival curves for the combination of the four genes (LAT, KRT17, MAGEC2, and RNASE2) methylation and expression. D, The expression of four genes (LAT, KRT17, MAGEC2, and RNASE2) increased significantly in both OSRC2 and 786O cell lines following treatment with 5‐Aza (8 µM) for 72 hours. We used P16 and CST6 as positive controls in these assays. *P < .05

In conclusion, our study explored the methylation‐driven and differently expressed genes in ccRCC and found four genes with potentials of prognostic biomarkers for ccRCC patients.

## CONFLICT OF INTEREST

The authors declare no potential conflict of interest.

## Supporting information

Figure S1. The relationship between gene expression and site methylation.

Table S1. Four genes (LAT, KRT17, MAGEC2, and RNASE2) were shown to be independent factors effecting survival.

## Data Availability

All the data and materials are available upon request from the authors.
