# Supplementary figures and images for: Four methylation‐driven genes may be prognostic biomarkers in clear cell renal carcinoma
Source: Clin Transl Med. 2020 Jun 4;10(2):e45. doi: 10.1002/ctm2.45 (PMC7403715; doi:10.1002/ctm2.45)

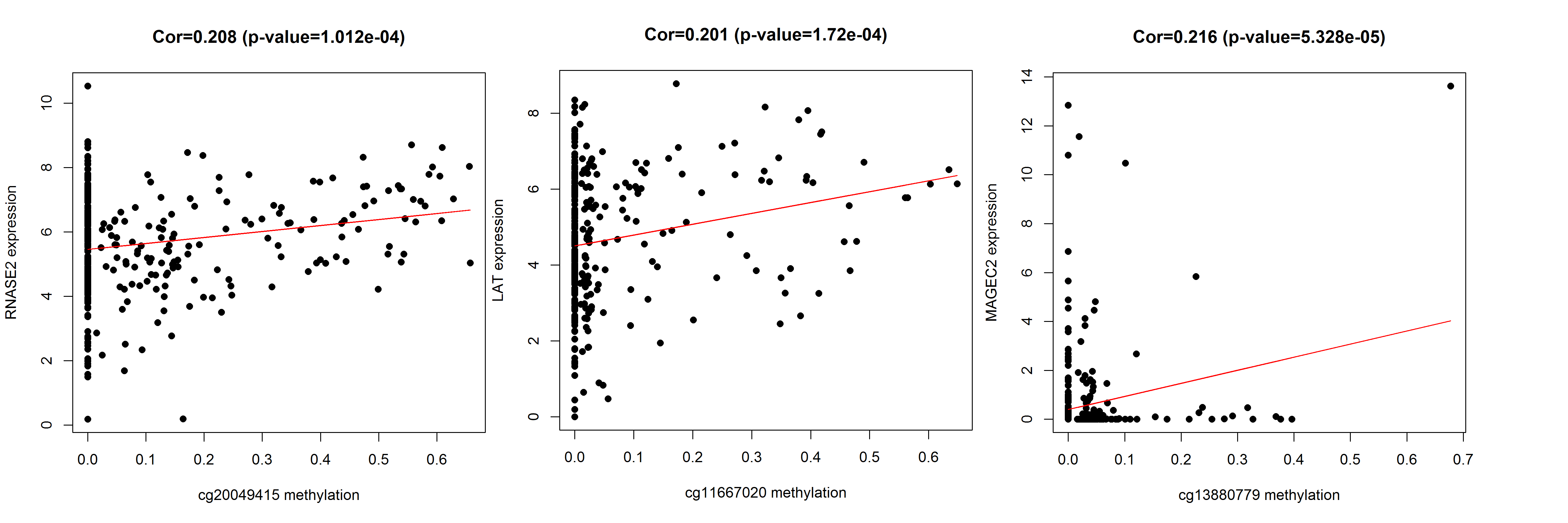

Supplement: Supplementary file 1 — Figure S1. The relationship between gene expression and site methylation. [file CTM2-10-e45-s001.tif]
