# Supplementary material for: Four methylation‐driven genes may be prognostic biomarkers in clear cell renal carcinoma
Source: Clin Transl Med. 2020 Jun 4;10(2):e45. doi: 10.1002/ctm2.45 (PMC7403715; doi:10.1002/ctm2.45)
Supplement: Supplementary file 2 — Table S1. Four genes (LAT, KRT17, MAGEC2, and RNASE2) were shown to be independent factors effecting survival. [file CTM2-10-e45-s002.doc]

Table S1. 4 genes (LAT, KRT17, MAGEC2 and RNASE2) were shown to be independent factors effecting survival.

| Gene | Coef | Exp(coef) | Se(coef) | Z | P |
| --- | --- | --- | --- | --- | --- |
| LAT | 4.24e-03 | 1.00e+00 | 1.07e-03 | 3.95 | 7.7e-05 |
| KRT17 | 3.21e-05 | 1.00e+00 | 1.41e-05 | 2.27 | 0.023 |
| MAGEC2 | 1.10e-03 | 1.00e+00 | 2.33e-04 | 4.73 | 2.2e-06 |
| RNASE2 | 1.74e-03 | 1.00e+00 | 5.64e-04 | 3.09 | 0.002 |
